# Soluble bone-derived osteopontin promotes migration and stem-like behavior of breast cancer cells

**DOI:** 10.1371/journal.pone.0177640

**Published:** 2017-05-12

**Authors:** Graciella M. Pio, Ying Xia, Matthew M. Piaseczny, Jenny E. Chu, Alison L. Allan

**Affiliations:** 1 Department of Anatomy & Cell Biology, Schulich School of Medicine & Dentistry, University of Western University, London, ON, Canada; 2 London Regional Cancer Program, London Health Sciences Centre, London, ON, Canada; 3 Department of Oncology, Schulich School of Medicine & Dentistry, University of Western University, London, ON, Canada; 4 Cancer Research Laboratories, Lawson Health Research Institute; London, ON, Canada; University of Alabama at Birmingham, UNITED STATES

## Abstract

Breast cancer is a leading cause of cancer death in women, with the majority of these deaths caused by metastasis to distant organs. The most common site of breast cancer metastasis is the bone, which has been shown to provide a rich microenvironment that supports the migration and growth of breast cancer cells. Additionally, growing evidence suggests that breast cancer cells that do successfully metastasize have a stem-like phenotype including high activity of aldehyde dehydrogenase (ALDH) and/or a CD44^+^CD24^-^ phenotype. In the current study, we tested the hypothesis that these ALDH^hi^CD44^+^CD24^-^ breast cancer cells interact with factors in the bone secondary organ microenvironment to facilitate metastasis. Specifically, we focused on bone-derived osteopontin and its ability to promote the migration and stem-like phenotype of breast cancer cells. Our results indicate that bone-derived osteopontin promotes the migration, tumorsphere-forming ability and colony-forming ability of whole population and ALDH^hi^CD44^+^CD24^-^ breast cancer cells in bone marrow-conditioned media (an *ex vivo* representation of the bone microenvironment) (p≤0.05). We also demonstrate that CD44 and RGD-dependent cell surface integrins facilitate this functional response to bone-derived osteopontin (p≤0.05), potentially through activation of WNK-1 and PRAS40-related pathways. Our findings suggest that soluble bone-derived osteopontin enhances the ability of breast cancer cells to migrate to the bone and maintain a stem-like phenotype within the bone microenvironment, and this may contribute to the establishment and growth of bone metastases.

## Introduction

Breast cancer is the most frequently diagnosed cancer among North American women, currently accounting for approximately 26% of all newly diagnosed cancer cases [1, 2]. Breast cancer’s high mortality rate (ranked second among women after lung cancer) is primarily due to the failure of conventional therapy to mitigate and eliminate metastatic disease. While breast cancer patients with localized disease at the time of diagnosis have an excellent (almost 90%) chance of long-term survival, a patient with metastatic disease has a mere 22% chance of surviving longer than ten years [1, 2].

Although lethal, metastasis is a surprisingly inefficient process, with the rate-limiting steps being the ability to initiate growth after extravasation into the secondary tissue and to maintain that growth into clinically detectable macrometastases [3]. Growing evidence suggests that breast cancer cells that can successfully initiate a primary tumor and traverse the entire metastatic cascade may be “stem-like” cells or so-called “cancer stem cells” (CSCs) because of their unique ability to self-renew and differentiate into a heterogenous tumor [4–7]. These stem-like breast cancer cells can be isolated using specific markers including a CD44^+^CD24^-^ phenotype and/or high aldehyde dehydrogenase activity (ALDH^hi^) [8, 9]. Our laboratory has pioneered functional characterization of these cells with regards to metastatic behavior, and were the first to report that stem-like ALDH^hi^CD44^+^CD24^-^ cells demonstrate increased proliferation, adhesion, migration and invasion *in vitro* and metastasis *in vivo* relative to their non-stem-like ALDH^low^CD44^-^CD24^+^ counterparts [10].

Clinically, breast cancer metastasizes in an organ-specific pattern to lymph nodes, lung, liver, bone and brain, with the bone being the most common site of metastasis [11–15]. Stephen Paget’s seminal “seed and soil” hypothesis, first proposed in 1889, posits that this organ-specific metastatic dissemination is mediated by crosstalk between a subset of cancer cells (the ‘seeds’) and specific organ microenvironments (the ‘soil’) [13]. A cancer cell’s altered genetic or molecular signature and unique cell surface receptors results in a predilection for certain organ microenvironments, and in turn a favorable niche provides conditions that promote metastatic development [16]. In support of this, a meta-analysis of published autopsy data [12] demonstrated that more bone metastases can be detected in breast cancer patients than would be expected by blood flow alone, indicating that the bone microenvironment is likely very important for metastatic dissemination and growth.

In the context of the CSC hypothesis, the bone microenvironment has been shown to be a rich stem cell niche [17]. Notably, studies have demonstrated that the majority of early-disseminated breast cancer cells found in the bone of breast cancer patients have a stem-like phenotype, an observation that complements the stem cell hypothesis of cancer metastasis [18]. Specifically, breast cancer cells with a CD44^+^ phenotype have been shown to have increased adherence to human bone marrow endothelial cells [19]. Furthermore, previous studies in our lab have demonstrated that both whole population and ALDH^hi^CD44^+^ breast cancer cells show enhanced migration towards bone marrow-conditioned media relative to control [20], although the soluble molecular factors that drive this require further investigation.

Within the bone microenvironment, the acidic phosphoglycoprotein osteopontin (OPN) is the most abundant non-collagenous extracellular matrix protein present, and as such, is a protein of interest when considering the bone as a favorable niche for breast cancer metastases. OPN mediates cell-matrix and cell-cell communication through interactions with a variety of cell surface receptors (i.e. CD44, α9β1, αvβ3 and αvβ5), leading to downstream activation of pathways that ultimately contribute to survival, migration, adhesion, proliferation, angiogenesis, and metastasis [21–24]. Clinically, OPN has been shown to have prognostic value, with observed correlations between plasma OPN levels, metastatic tumor burden and survival in breast cancer patients [25–27]. Although extensive biological research has focused on investigating the functional implications of tumor cell-derived OPN in cancer, the role of bone-derived OPN in the metastatic progression of breast cancer remains poorly understood.

In the current study, we therefore tested the hypothesis that soluble bone-derived OPN plays an important role in the metastatic behavior of breast cancer cells. We demonstrate that bone-derived OPN promotes the migration and stem-like properties of breast cancer cells via the cell surface receptor CD44 and RGD-dependent cell surface integrins, resulting in activation of WNK-1 and PRAS40. Our findings suggest that soluble bone-derived OPN enhances the ability of breast cancer cells to migrate to bone and maintain a stem-like phenotype within the bone microenvironment, and this may contribute to the establishment and growth of bone metastases.

## Materials and methods

### Cell culture and reagents

MDA-MB-231 human breast cancer cells [28] were obtained directly from American Type Culture Collection (cat #ATCC-HTB-26; Manassas, VA) and maintained in DMEM/F12 + 10% fetal bovine serum (FBS). SUM159 human breast cancer cells [29] were obtained directly from Asterand Inc. (cat # SUM-159PT, Detroit, MI) and maintained in HAMS:F12 + 5% FBS + 5 μg/mL insulin + 1 μg/mL hydrocortisone + 10mM HEPES. Media/supplements were from Invitrogen (Carlsbad, CA) and FBS was from Sigma-Aldrich (St. Louis, MO). Cell lines were authenticated via third-party testing (IDEXX, Columbia, MO).

### Bone-marrow conditioned media

Mice were used for this study. All studies were carried out in strict accordance with the recommendations of the Canadian Council for Animal Care. The protocol was approved by the Animal Care Committee at the University of Western Ontario (protocol# 2009–064). Euthanasia was performed with the use of CO_2_ and all efforts were made to minimize suffering. Bone marrow-conditioned media (BMCM) was generated as previously described [20] using bones harvested from healthy female nude mice. Briefly, healthy female nude mice (6–12 weeks old; Hsd: Athymic Nude-Foxn1^nu^; Harlan Sprague-Dawley, Indianapolis, IN) were euthanized and bone marrow was collected by flushing femur cavities. Aspirates were dissociated into single cells by pipetting and cells were washed and plated in DMEM + 10% FBS + penicillin/streptomycin (pen/strep). Resulting adherent bone marrow stromal cells (BMSCs) were passaged 2–3 times, washed, and exposed to DMEM/F12 + Mito^+^™ + pen/strep. Conditioned media was collected after 72 hours and stored at -20°C. To account for mouse-to-mouse variability, BMCM from multiple mice was pooled prior to use.

### Protein array analysis

To identify soluble factors present within BMCM, RayBio® AAM-BLM-1 label-based mouse antibody arrays were used to simultaneously assess of expression of 308 soluble murine target proteins (RayBiotech Inc., Norcross, GA) as described previously [20]. Results were visualized using chemiluminescence and film exposure (CL-Xposure Film, Pierce). Densitometric analysis was conducted using Image J with the MicroArray Profile Macro and results (N = 3/media condition) were analyzed using the RayBiotech analysis tool for AAM-BLM-1 as described previously [20]. To identify potential phosphorylation changes in human breast cancer cells in response to BMCM, MDA-MB-231 human cells were incubated in basal media, BMCM, or BMCM depleted of OPN and cell lysates were harvested after 2 hours. Protein concentrations were determined with a DC protein assay (BioRad). Cell lysates were incubated with the Human Phospho-Kinase Array membranes (ARY003B, R&D Systems) overnight at 4°C. Biotinylated detection antibodies were applied and membranes were visualized using chemiluminescence. Densiotometric analysis was performed using the Protein Array Analyzer for ImageJ (N = 3/ media condition).

### Immunodepletion

Osteopontin was immunodepleted from BMCM using a rat anti-mouse OPN-specific antibody (R&D Systems, Burlington, ON). Antibodies were incubated for 20 minutes at room temperature (RT) with Dynabeads Protein G (8 μg OPN-specific antibody per mg of beads; Novex/Life Technologies, Oslo). Bead-antibody complexes were then incubated with BMCM for 30 minutes at RT. Resulting bead-antibody-antigen complexes were removed from BMCM using a DynaMag-2 magnet (Novex/Life Technologies). The concentration of OPN in depleted BMCM was assessed by Quantikine ELISA kits specific for mouse OPN (R&D Systems). Negative controls included BMCM exposed to beads only (no antibody). GST-tagged human OPN (GST-hOPN; a kind gift from Dr. Ann Chambers, London Regional Cancer Program, London, ON [30]) was used for rescue experiments at the same concentration that was originally depleted from BMCM.

### Cell migration assays

Transwells^®^ (6.5 mm, 8 μm pore size; Falcon, Corning, NY) were coated with 6 μg of gelatin per well. Osteopontin-depleted, non-depleted BMCM, or basal media was placed in 24-well dishes (n = 3 per condition). MDA-MB-231 or SUM-159 human breast cancer cells (5 x 10^4^ cells per well) were plated on top of the gelatin-coated Transwells^®^ and inserted into 24-well dishes. In experiments involving functional blocking of CD44 or RGD, cells were incubated for 30 minutes at RT with rat anti-human CD44 antibody (10 μg per 5 x 10^5^ cells; Calbiochem, Mississauga, ON) or an RGD-sequence specific peptide (50 μg per 5 x 10^5^ cells for MDA-MB-231 breast cancer cells, 100 μg per 5 x 10^5^ cells for SUM-159 breast cancer cells; Sigma-Aldrich). For PI3K/Akt inhibitor experiments, breast cancer cells were first serum-starved for 18 hours, harvested and pretreated for 1 hour with 20 μM of either LY294002 or Triciribine (Millipore, Temecula, CA) or an equivalent concentration of vehicle (DMSO) prior to the migration assay. After 18 hours, Transwells^®^ were fixed and non-migrated cells were removed from the inner surface and migrated cells on the lower surface were stained with DAPI. Five high-powered fields (HPFs) of view were counted for each membrane using ImageJ [National Institutes of Health (NIH), Bethesda, MD] software. Results are expressed as a fold-increase from negative control (N = 3).

### Sphere-limiting dilution assays (SLDA) and colony-forming assays

MDA-MB-231 human breast cancer cells were seeded into 96-well plates for colony-forming assays (Corning, Lowell, Massachusetts) or 96-well Ultra-Low Attachment plates (Corning) for the sphere-limiting dilution assay (SLDA) in a serial-diluted fashion ranging from 1000–0.001 cells/well. For functional blocking experiments, anti-CD44 antibody or RGD-sequence specific peptide were used as described for the migration assays. Osteopontin-depleted, non-depleted BMCM, or basal media was added to the wells and cells were cultured for 5 days. At the end of the assay, each well was scored for the presence or absence of colonies or tumorspheres (N = 3 each) using L-Calc™ Software (Stem Cell Technologies, Vancouver, BC).

### Fluorescence-activated cell sorting (FACS) and flow cytometry

ALDH^hi^CD44^+^CD24^-^ and ALDH^lo^CD44^-^CD24^+^ cells subpopulations were isolated from the MDA-MB-231 human breast cancer cell line as described previously [10, 31]. Briefly, cells were concurrently labeled with 7-amino-actinomycin D (7-AAD), ALDEFLUOR^TM^ assay kit (StemCell Technologies; Vancouver, BC) and fluorescently-conjugated antibodies including anti-CD44 (clone IM7) conjugated to allophycocyanin (APC) and anti-CD24 (clone ML5) conjugated to phycoerytherin (PE) (BD Biosciences). ALDH activity was used as the primary sort criteria (top ~20% = ALDH^hi^; bottom ~20% = ALDH^low^) and CD44^+^CD24^-^ phenotype as the secondary sort criteria (top ~10% gated on ALDH^hi^; bottom ~10% gated on ALDH^low^). Cell viability was assessed by 7-AAD staining during cell sorting, and confirmed by trypan blue exclusion post-sorting. FACS-isolated cells were used immediately for *in vitro* assays.

For flow cytometry analysis, MDA-MB-231 and SUM159 human breast cancer cells were grown to 80% confluence in normal growth media, harvested and resuspended at 1 x 10^6^ cells/ml. Cells were then incubated with phycoerytherin (PE)-conjugated CD44 (BD Biosciences, San José, CA), fluorescein isothiocyanate (FITC)-conjugated αvβ3 (R&D Systems), Alexafluor (AF)-488-conjugated αvβ5 (R&D Systems), AF-488-conjugated β1 (R&D Systems) or AF-488-conjugated α9β1 (R&D Systems) antibodies for 1 hour at 4°C. Negative controls included cells only (no antibody) and cells incubated with an isotype-matched IgG-control. Samples were analyzed on a Beckman-Coulter EPICS XL-MCL flow cytometer.

### Immunoblotting

MDA-MB-231 human cells were incubated in basal media, BMCM, or BMCM depleted of OPN and cell lysates were harvested after 2 hours. Protein concentrations were determined with a DC protein assay (BioRad). Immunoblotting was used to assess the expression of total PRAS40 (anti-human PRAS40 [Clone 660928], R&D Systems), phosphorylated PRAS40 (anti-human Phospho-PRAS40 [T246] [Clone 760502], R&D Systems), total WNK-1 (anti-human polyclonal Ab, Cell Signaling), or phosphorylated WNK-1 (anti-human Phospho-WNK1 [T60] polycolonal, R&D Systems). β-actin was used as a loading control (anti-human anti-ACTB [SAB2108641], Sigma).

### siRNA targeting of WNK1 and PRAS40

For knockdown of WNK1 and PRAS40, ON-TARGETplus SMART pools of 4 specific or control small interfering RNAs (siRNA) (Dharmacon Thermo Scientific, Lafayette, CO) were used in combination with transient transfection of MDA-MB-231 human breast cancer cells. All siRNAs were suspended in sterile RNAse-free water at a concentration of 25 μM. Scrambled control, WNK1, or PRAS40 siRNA pools (6ul) and 10ul Lipofectamine RNAiMAX reagent (Invitrogen) were diluted into serum-free Opti-MEM media (Invitrogen) and incubated for 20 min at room temperature before addition to MDA-MB-231 cells (50% confluency) in 60 mm culture dishes. After 48 hours of incubation at 37°C, 5% C0_2_, cells were harvested, assessed for knockdown efficiency by immunoblotting and used for migration assays as described above.

### Data analysis

*In vitro* experiments were performed a minimum of three times with three technical replicates within each experiment. Unless otherwise noted, data are presented as mean ± SEM. Statistical analysis was performed using GraphPad Prism 6.0 (GraphPad Software, San Diego, CA) using one-way analysis of variance (ANOVA) with Tukey’s or Bonferroni’s post-hoc tests. In all cases, values of p<0.05 were classified as being statistical significant.

## Results

### Soluble OPN is produced in the bone microenvironment and enhances breast cancer cell migration

**Fig 1:**
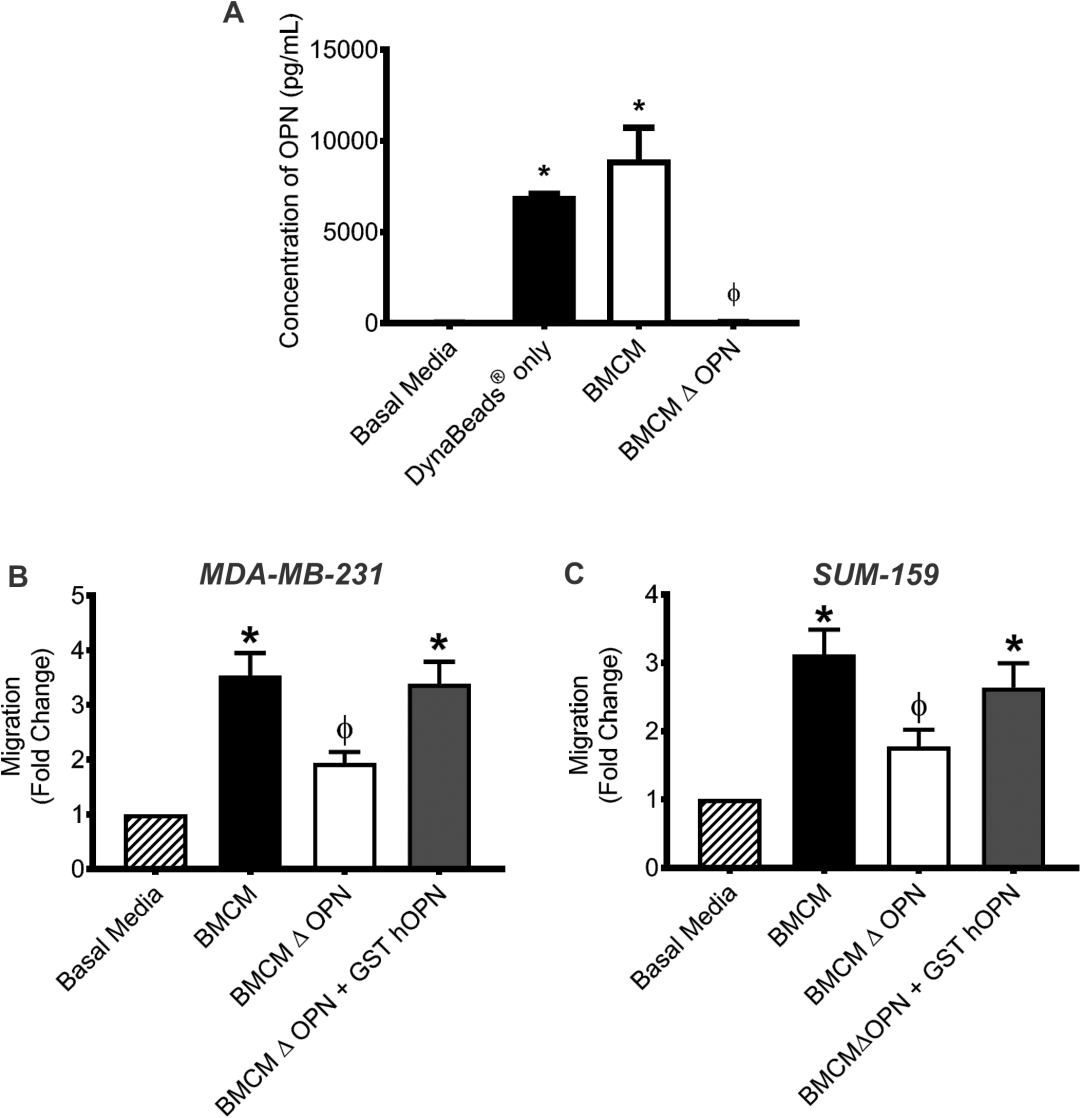
Soluble OPN is produced in the bone microenvironment and enhances human breast cancer cell migration. (A) Goat anti-mouse OPN primary antibody was incubated for 20 min at RT with DynaBeads^®^ Protein G prior to incubation with BMCM for 30 min at RT. The beads-antibody-antigen complex was removed with a DynaMag™-2 magnet. Concentration of OPN in BMCM, BMCM with beads only (no antibody) and BMCM depleted of OPN (ΔOPN) was assessed by ELISA. Data are presented as mean ± SEM (N = 3). (B) MDA-MB-231 and (C) SUM-159 cells were subjected to transwell migration assays (5 x 10^4^ cells/well; 8μm pore size) using basal media (DMEM/F12 + Mito^+^), BMCM, BMCM depleted of OPN (ΔOPN) or BMCM ΔOPN rescued with GST-hOPN. Plates were incubated at 37°C, 5% CO_2_ for 18 hr, fixed and stained. Five high-powered fields of view (HPF) were captured per transwell and migrated cells were analyzed. Data are presented as mean ± SEM (N = 3; fold-change from negative control basal media). * = significantly different than basal media; ϕ = significantly different than BMCM (P≤0.05).

An initial investigation of potential soluble factors present in the bone microenvironment was carried out using protein array analysis of bone marrow-conditioned media (BMCM) as a model system [20]. Of the proteins identified in BMCM, several were found to be associated with metastasis, including the soluble phosphoprotein osteopontin (OPN) (S1 Table). In the current study, we chose to focus our work on OPN because of its established function in both the normal bone microenvironment and during breast cancer metastasis [21–24]. ELISA analysis demonstrated that that BMCM contains significant amounts of bone-derived OPN compared to basal media (P≤0.05; Fig 1A). To assess the effect of bone-derived OPN on human breast cancer cell migration, OPN was immunodepleted from BMCM. Transwell^TM^ migration assays were then used to assess the migration of MDA-MB-231 and SUM-159 human breast cancer cells to basal media, BMCM and BMCM depleted of OPN. Both cell lines exhibited increased migration toward BMCM relative to basal media (P≤0.05; Fig 1B and 1C) and exhibited significantly decreased migration to BMCM depleted of OPN, back down to levels comparable to basal media (P≤0.05; Fig 1B and 1C). To validate that bone-derived OPN was specifically responsible for the observed effects on breast cancer cell migration, recombinant human OPN (GST-hOPN) was added back into BMCM depleted of OPN at the same concentration that was originally depleted. The addition of GST-hOPN to BMCM rescued the migratory effect on both MDA-MB-231 and SUM-159 cells, causing cells to migrate at similar levels as non-depleted BMCM (Fig 1B and 1C). These results demonstrate that bone-derived OPN enhances breast cancer cell migration towards BMCM.

### Bone-derived OPN enhances the migration and stem-like behavior of ALDH^hi^CD44^+^CD24^-^ breast cancer cells

**Fig 2:**
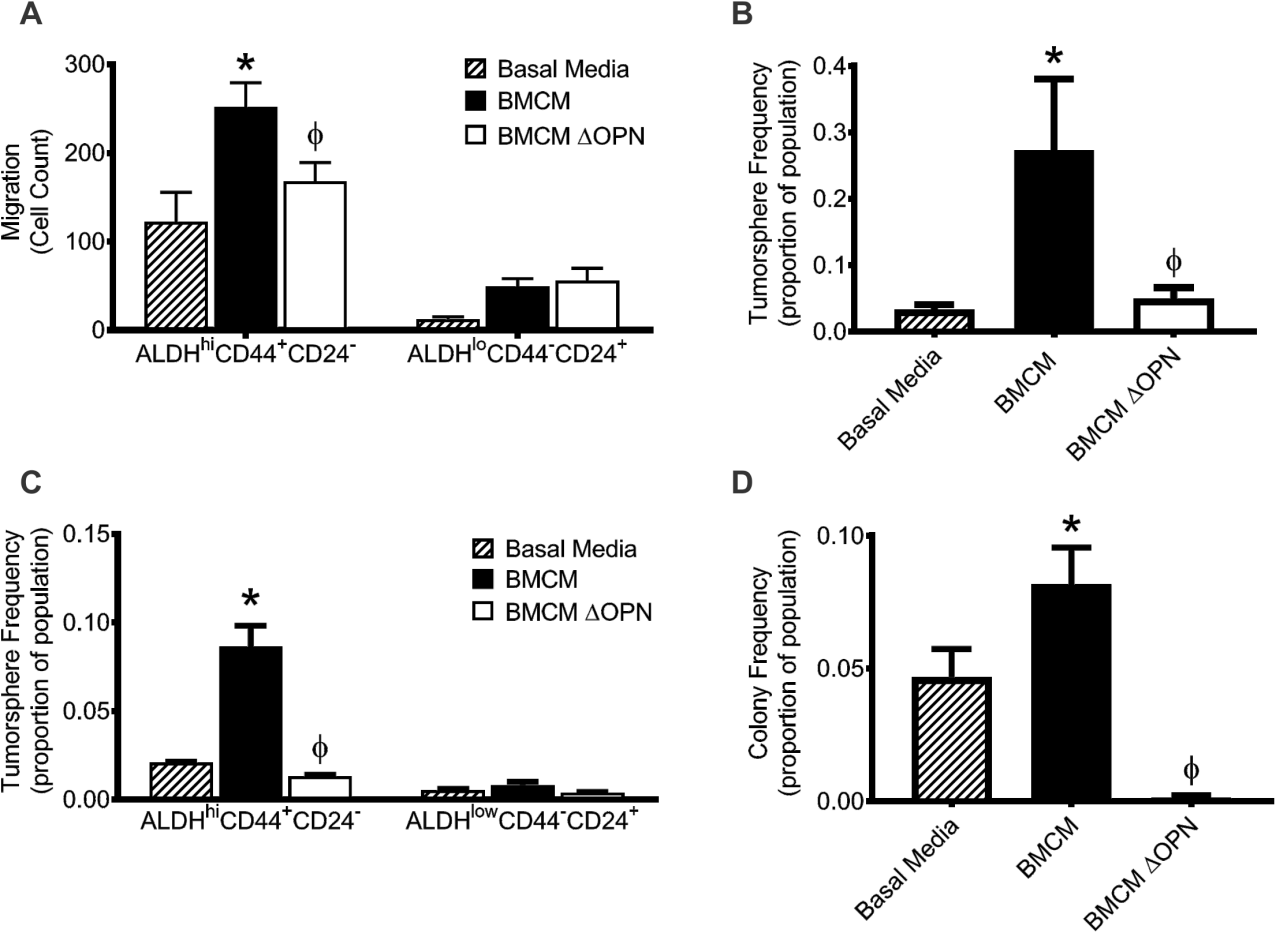
Bone-derived OPN enhances the migration and stem-like behavior of ALDH^hi^CD44^+^CD24- breast cancer cells. (A) ALDH^hi^CD44^+^CD24^-^ and ALDH^lo^CD44^-^CD24^+^ cell subpopulations were isolated from the MDA-MB-231 human breast cancer cell line by FACS and subjected to transwell migration assays (5 x 10^4^ cells/well; 8μm pore size) using basal media (DMEM/F12 + Mito^+^), BMCM, or BMCM depleted of OPN (ΔOPN). Plates were incubated at 37°C, 5% CO_2_ for 18 hr, fixed and stained. Five high-powered fields of view (HPF) were captured per transwell and migrated cells were analyzed. (B) Whole population or (C) ALDH^hi^CD44^+^CD24^-^ and ALDH^lo^CD44^-^CD24^+^ subpopulations isolated from the MDA-MB-231 breast cancer cell line were plated in a limiting dilution fashion on 96-well ultra-low attachment plates for 7 days in basal media, BMCM, or BMCM ΔOPN and subjected to sphere-forming assays. (D) Whole population MDA-MB-231 cells were plated in a limiting dilution fashion on normal 96-well plates for 7 days in basal media, BMCM, or BMCM ΔOPN and subjected to colony-forming assays. Data are presented as mean ± SEM (N = 3). * = significantly different than basal media; ϕ = significantly different than BMCM (P≤0.05).

As previously discussed, “stem-like” ALDH^hi^CD44^+^ CD24^-^ breast cancer cells show enhanced metastasis to multiple different organs, including bone [7, 10]. We have previously shown that this subpopulation is found within the MDA-MB-231 cell line and shows increased migration to BMCM relative to ALDH^lo^CD44^-^CD24^+^ cells [20]. Thus, we wanted to investigate if bone-derived OPN influenced the migration of this population. Using FACS, ALDH^hi^CD44^+^CD24^-^ and ALDH^lo^CD44^-^CD24^+^ subpopulations were isolated from the MDA-MB-231 cell line using the strategy outlined in S1 Fig and used in migration assays in combination with basal media, BMCM, or BMCM depleted of OPN. The ALDH^hi^CD44^+^ CD24^-^ subpopulation demonstrated significantly increased migration toward BMCM compared to basal media, an effect that was abrogated when OPN was depleted from BMCM (P≤0.05; Fig 2A). In contrast, ALDH^lo^CD44^-^CD24^+^ cells did not display significantly increased migration towards either BMCM or BMCM depleted of OPN compared to basal media (P>0.05; Fig 2A).

Next, we used a sphere limiting dilutions assay (SLDA) to assess the effect of bone-derived OPN on the tumorsphere-forming capacity of breast cancer cells. Whole population MDA-MB-231 cells demonstrated increased tumorsphere-forming capacity in BMCM compared to basal media (P≤0.05; Fig 2B). This tumorsphere-forming ability decreased when BMCM was depleted of OPN (P≤0.05; Fig 2B), suggesting that bone-derived OPN supports this capacity in MDA-MB-231 cells. Additionally, the tumorsphere-forming ability of ALDH^hi^CD44^+^ CD24^-^ cells was reduced in the presence of BMCM depleted of OPN relative to BMCM (P≤0.05; Fig 2C), indicating that this cell subpopulation is responsible for the tumorsphere-forming capacity of MDA-MB-231 cells in BMCM and that bone-derived OPN supports their stem-like phenotype. We also investigated the influence of bone-derived OPN on the colony-forming ability of MDA-MB-231 cells. Whole population MDA-MB-231 cells show increased colony-forming abilities in BMCM compared to basal media (P≤0.05; Fig 2D). When cells were exposed to BMCM depleted of OPN, their colony-forming capacity was significantly reduced (P≤0.05; Fig 2D), suggesting that bone-derived OPN also plays a role in the colony-forming ability of breast cancer cells.

### Bone-derived OPN interacts with CD44 and RGD-dependent integrins to enhance migration

OPN is known to influence multiple steps in the metastatic cascade through the cell surface receptor CD44, as well as multiple different cell surface integrins; including αvβ1, α9β1 αvβ3 and αvβ5 [21]. Thus, we first evaluated the expression of CD44, β1, α9β1 αvβ3 and αvβ5 on the MDA-MB-231 and SUM-159 cell lines. Flow cytometry indicated that both cell lines are positive for the expression of CD44 and these four cell surface integrins (S2 and S3 Figs). Next, we investigated the role of these cell surface proteins in the migration of breast cancer cells using an anti-CD44 functional blocking antibody or an Arg-Gly-Asp (RGD) peptide. OPN contains an RGD sequence that is recognized by β1, α9β1 αvβ3 and αvβ5 integrins; therefore incubating the cells with the RGD-sequence specific peptide prior to use in functional assays should block the recognition of OPN via the RGD sequence [30, 32].

**Fig 3:**
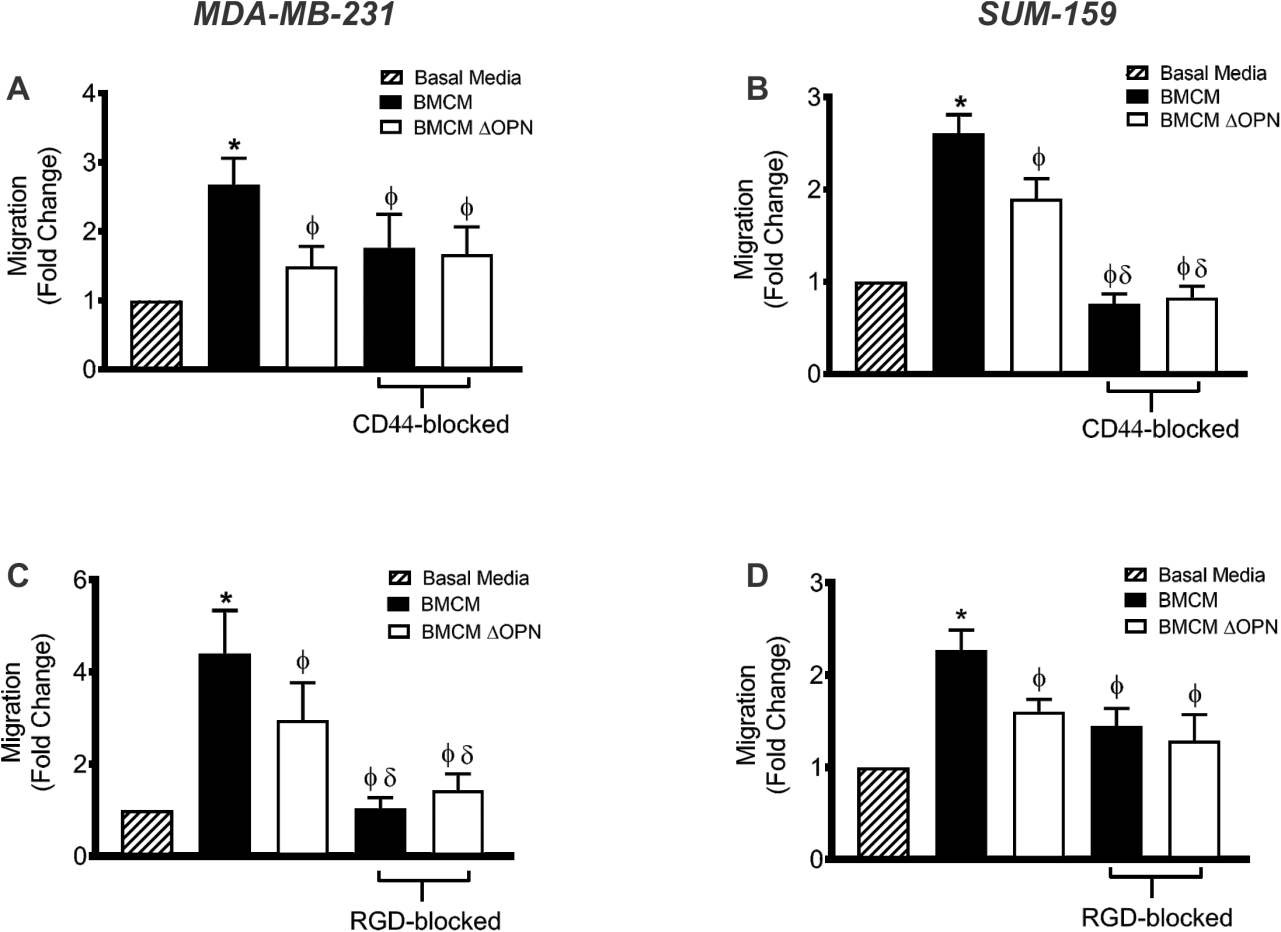
Bone-derived OPN interacts with CD44 and RGD-dependent integrins to enhance breast cancer cell migration. MDA-MB-231 and SUM-159 human breast cancer cells were blocked with an anti-CD44 antibody (A, B) or an RGD sequence-specific blocking peptide (C, D) for 30 min and subjected to transwell migration assays (5 x 10^4^ cells/well; 8μm pore size) using basal media (DMEM/F12 + Mito^+^), BMCM, or BMCM depleted of OPN (ΔOPN). Plates were incubated at 37°C, 5% CO_2_ for 18 hr, fixed and stained. Five high-powered fields of view (HPF) were captured per transwell and migrated cells were analyzed. Data are presented as mean ± SEM (N = 3; fold change from negative control of basal media). * = significantly different than basal media; ϕ = significantly different than non-blocked BMCM, δ = significantly different than non-blocked BMCM ΔOPN (P≤0.05).

We observed that blocking CD44 on the MDA-MB-231 and SUM-159 cells significantly decreased migration to BMCM compared to migration of untreated cells (P≤0.05; Fig 3A and 3B). The migration of CD44-blocked MDA-MB-231 cells to BMCM was only reduced to a level similar to untreated cells exposed to BMCM depleted of OPN, suggesting that CD44 specifically contributes to the effect of bone-derived OPN on breast cancer cell migration (P>0.05; Fig 3A). In contrast, CD44-blocked SUM-159 cell migration to BMCM was significantly lower than untreated SUM-159 cell migration to BMCM depleted of OPN (P≤0.05; Fig 3B). This suggests that CD44 may mediate the interaction of SUM-159 with bone-derived OPN as well as other soluble factors within BMCM.

Our results also indicate that treatment of MDA-MB-231 and SUM-159 breast cancer cells with a RGD-blocking peptide significantly reduces their migration to BMCM relative to untreated cells (P≤0.05; Fig 3C and 3D). RGD-blocked MDA-MB-231 cells showed significantly decreased migration to BMCM compared to untreated cells exposed to BMCM depleted of OPN (P≤0.05; Fig 3C). These results suggest that RGD-dependent integrins may mediate the interaction of MDA-MB-231 with bone-derived OPN as well as other soluble factors within the BMCM. In contrast, RGD-blocked SUM-159 cells showed similar migratory levels to BMCM as untreated cells to BMCM depleted of OPN, suggesting that RGD-dependent cell surface integrins specifically mediate the interaction of SUM-159 cells with bone-derived OPN (P>0.05; Fig 3D).

### Promotion of breast cancer cell stem-like behavior by bone-derived OPN is mediated through CD44 and RGD-dependent integrins

**Fig 4:**
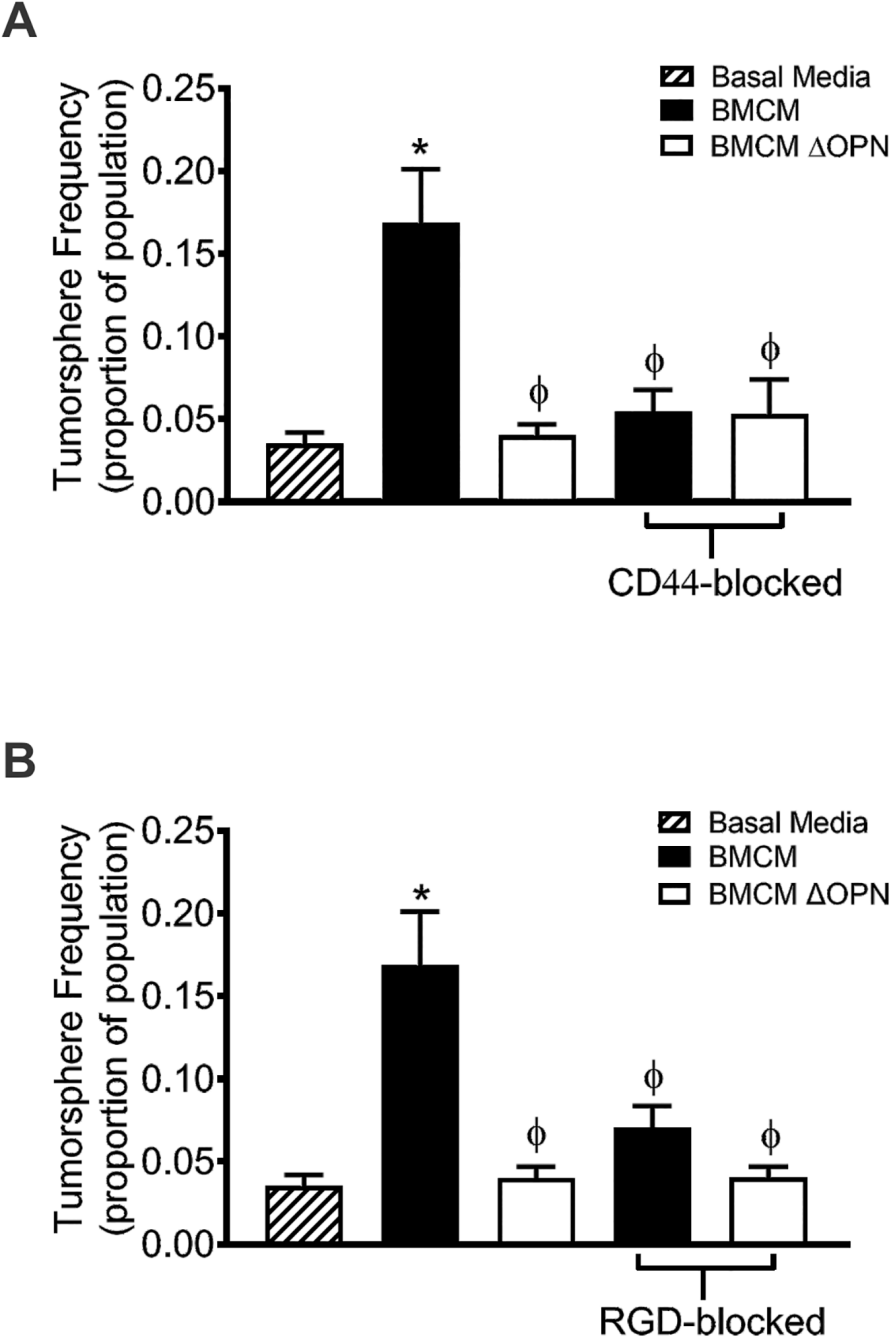
Breast cancer cell stem-like behavior is mediated by bone-derived OPN through CD44 and RGD-dependent integrins. MDA-MB-231 human breast cancer cells were blocked with (A) an anti-CD44 antibody or (B) an RGD sequence-specific blocking peptide for 30 minutes prior to plating in a limiting dilution fashion on ultra-low adhesion 96-well plates for 7 days in basal media (DMEM/F12 + Mito^+^), BMCM or BMCM ΔOPN in the sphere limiting dilution assay (SLDA). Data are presented as mean ± SEM (N = 3; fold change from negative control of basal media). * = significantly different than basal media; ϕ = significantly different than non-blocked BMCM (P≤0.05).

Given that CD44 and RGD-dependent integrins mediate the interaction between breast cancer cells and bone-derived OPN to influence migration to BMCM, we wanted to explore if these cell surface receptors also influence the stem-like phenotype of breast cancer cells. The anti-CD44 blocking antibody and RGD-sequence specific peptide were used to block MDA-MB-231 in the SLDA. Both CD44-blocked and RGD-blocked MDA-MB-231 cells showed decreased tumorsphere-forming capacity when exposed to BMCM and BMCM depleted of OPN compared to untreated cells exposed to BMCM depleted of OPN (P≤0.05; Fig 4A and 4B). This suggests that both CD44 and RGD-dependent integrins can mediate the tumorsphere-forming capacity of breast cancer cells through interactions with OPN.

### Bone-derived OPN induces phosphorylation of WNK1 and PRAS40

**Fig 5:**
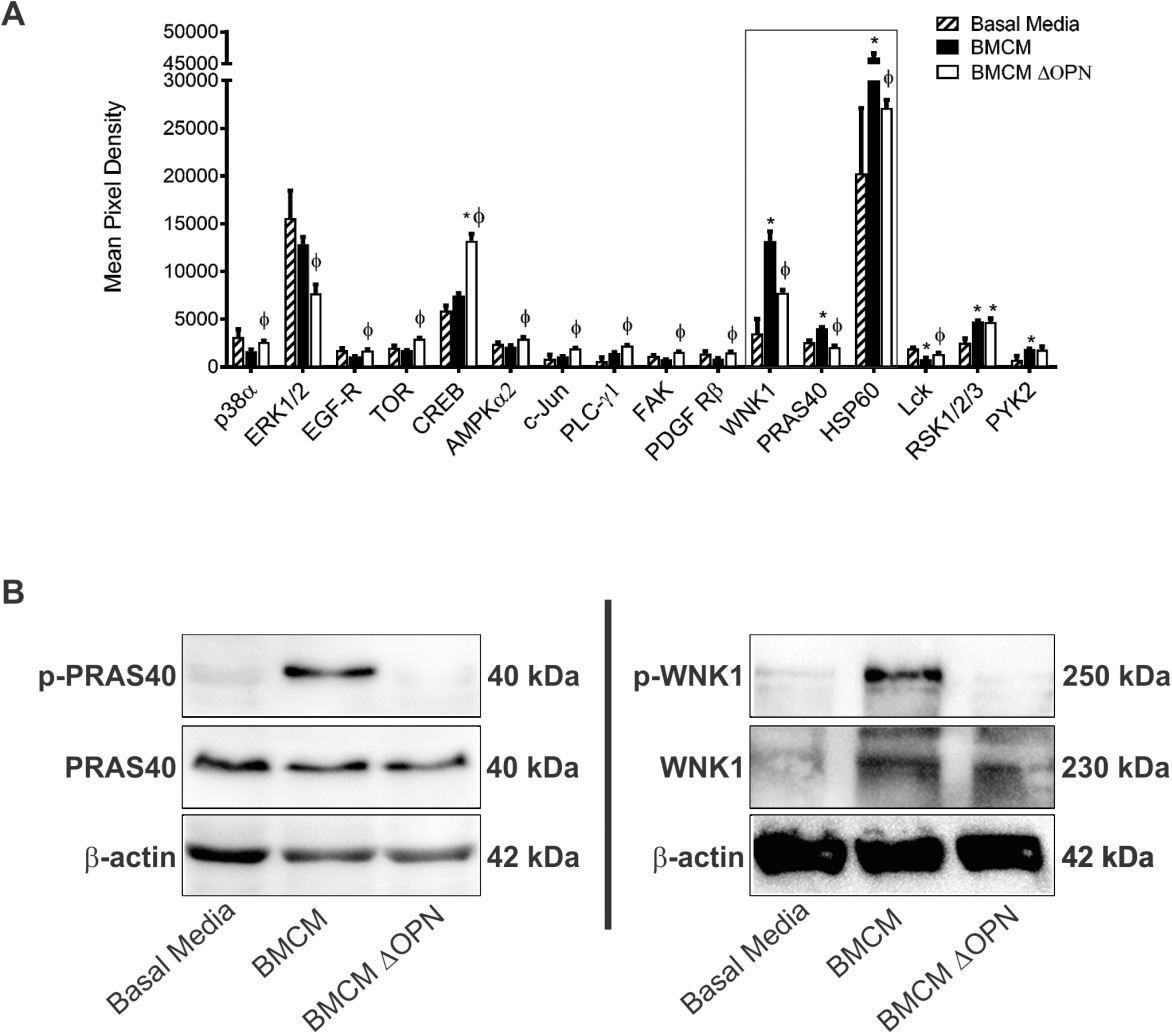
Bone-derived OPN induces phosphorylation of WNK1 and PRAS40. MDA-MB-231 human breast cancer cells were exposed to basal media, BMCM and BMCM depleted of OPN (BMCM ΔOPN) for 2 hours, and cell lysates were harvested. (A) Cell lysates were assessed using with Human Phospho-Kinase Array membranes (ARY003B, R&D Systems) overnight at 4°C. Biotinylated detection antibodies were applied and membranes were visualized using chemiluminescence. Densitometry analysis was performed using the Protein Array Analyzer for ImageJ (N = 3 for each media condition). * = significantly different than basal media; ϕ = significantly different than BMCM (P≤0.05). Only proteins with significantly different phosphorylation or expression between at least two treatments are shown. Proteins within the rectangular box demonstrated a similar pattern of phosphorylation to each other (increase in response to BMCM, with a subsequent decrease upon depletion of bone-derived OPN). (B) Phosphorylation patterns for PRAS40 and WNK1 were successfully validated using immunoblotting (N = 3).

Thus far, we have shown that bone-derived OPN influences the migratory ability and stem-like phenotype of breast cancer cells via CD44 and RGD-dependent integrins. Considering this, we wanted to investigate the effect of bone-derived OPN on downstream pathways within breast cancer cells. MDA-MB-231 cells were exposed to basal media, BMCM, or BMCM depleted of OPN. Cell lystates were collected after 2 hours and assessed using human phospho-kinase arrays. We observed that phosphorylation of WNK1, PRAS40, and HSP60 increased after exposure to BMCM, and this phosphorylation was abrogated when OPN was depleted (Fig 5A). We were able to validate these findings for PRAS40 (Fig 5B) and WNK1 (Fig 5C) using immunoblotting, but not HSP60. These findings indicate that bone-derived OPN leads to phosphorylation of WNK1 and PRAS40 and suggests that activation of these pathways may be a potential contributing mechanism underlying the functional role of bone-derived OPN in the malignant behavior of breast cancer cells.

### WNK1 and PRAS40-related pathways play a role in BMCM-mediated migration of breast cancer cells

**Fig 6:**
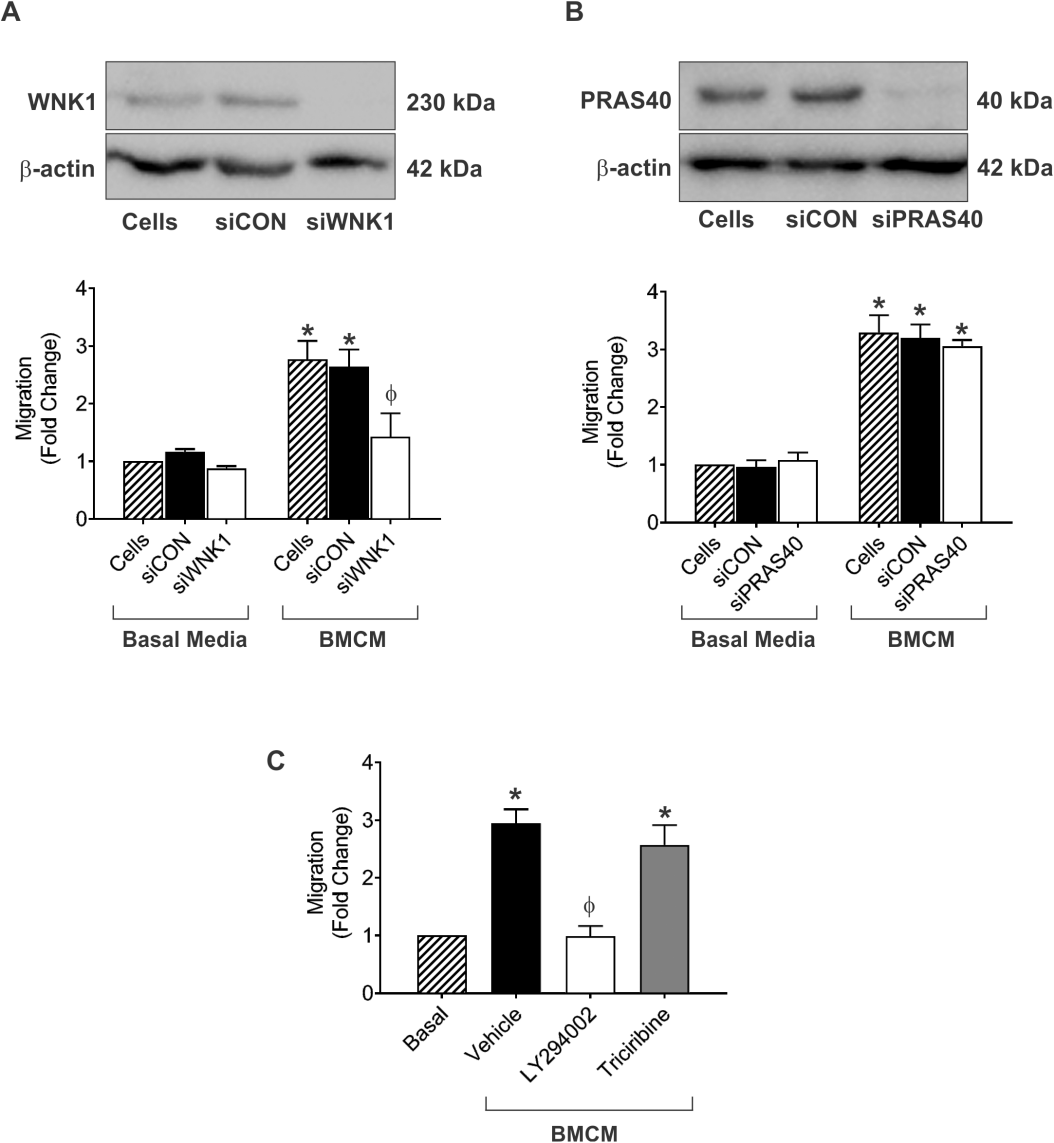
WNK1 and PRAS40-related pathways play a role in BMCM-mediated migration of breast cancer cells. (A, B) MDA-MB-231 human breast cancer cells were subjected to siRNA knockdown of WNK1 (A) or PRAS40 (B). Knockdown was confirmed by immunoblotting (top panels) prior to carrying out transwell migration assays (bottom panels; 5 x 10^4^ cells/well; 8μm pore size) using basal media (DMEM/F12 + Mito^+^) or BMCM. (C) MDA-MB-231 human breast cancer cells were serum-starved and treated with 20μM of PI3K inhibitor (LY294002) or Akt inhibitor (Triciribine) or an equivalent concentration of vehicle (DMSO) for 1 hour before being subjected to transwell migration assays (5 x 10^4^ cells/well; 8μm pore size) using basal media (DMEM/F12 + Mito^+^) or BMCM. Plates were incubated at 37°C, 5% CO_2_ for 18 hr, fixed, and stained. Five high-powered fields of view (HPF) were captured per transwell and migrated cells were analyzed. Data are presented as mean ± SEM (N = 3; fold change from negative control of basal media). * = significantly different than basal media; ϕ = significantly different than BMCM + siCON (A, B) or BMCM + vehicle (C) (P≤0.05).

To explore the potential role of these factors further, we directly targeted WNK1 and PRAS40 using siRNA. We observed that knockdown of WNK1 resulted in a significant reduction of breast cancer cell migration in response to BMCM relative to a non-specific siRNA control (Fig 6A; P≤0.05). In contrast, knockdown of PRAS40 had no effect on BMCM-mediate breast cancer cell migration (Fig 6B). Since PRAS40 is downstream of both PI3K and Akt [33], we hypothesized that its activation and effect on migration was indirectly related to the activity of one of these pathways. We observed that treatment with the PI3K inhibitor LY294002 resulted in a significant reduction of breast cancer cell migration in response to BMCM (Fig 6C; P≤0.05), whereas treatment with the Akt inhibitor Triciribine or a vehicle control had no effect (Fig 6C).

## Discussion

Approximately 85% of all breast cancer patients who die from their disease are expected to show metastasis to the bone [34]. These bone metastases pose an extreme burden for breast cancer patients, resulting in pain, fractures and hypercalcaemia, and the current clinical management for metastatic patients fails to prevent or cure these skeletal lesions [34].

The frequency of bone metastases in patients suggests that the bone microenvironment is conducive to the migration and growth of breast cancer cells [35]. Many previous studies have focused on the contribution of breast cancer cell-specific genes, receptors and secreted factors that contribute to the establishment of bone metastases in breast cancer patients. For example, there exists in the literature a wide breadth of knowledge on the effect of tumor-derived OPN in multiple steps of the metastatic cascade [24, 36–46]. However, much less research has been conducted on the effect of host-derived OPN in the tumor microenvironment. The current study focused on elucidating the role of bone-derived OPN on the migration, stem-like behavior and downstream signaling response of breast cancer cells.

We first demonstrated that OPN promotes the migration of breast cancer cells toward bone-marrow conditioned media. Taken together with previous work demonstrating that OPN-deficient mice display less colonization and growth of injected melanoma cells in bone than wild-type mice [47], this data suggests that bone-derived OPN increases breast cancer cell migration to and colonization in bone, thus promoting breast cancer bone metastases. Interestingly, both human breast cancer cell lines used in this study (MDA-MB-231 and SUM-159) displayed reduced migration towards BMCM depleted of OPN compared to non-depleted BMCM. However, levels of migration toward BMCM depleted of OPN were still significantly higher than migration toward basal media, suggesting that other factors within the BMCM may be contributing to the migration of these cancer cells. We identified other soluble factors present within the BMCM that could be responsible for the observed effect, including matrix-metalloproteinase-14 (MMP-14; MTI-MMP) and intracellular adhesion molecule-1 (ICAM-1), and these require further investigation.

Our results also suggest a novel function for bone-derived OPN in promoting the tumorsphere and colony-forming abilities of breast cancer cells. Sphere-limiting dilution assays (SLDA) and colony-forming assays were used assess the number of tumorsphere-initiating cells present in populations and the ability of cells to proliferate into colonies at very low numbers, which taken together can help evaluate the “stemness” of a population *in vitro* [48].

In healthy tissues, normal stem cells reside in specialized niches that influence their ability to remain quiescent, self-renew or differentiate via cell-to-cell interactions and/or via response to various secreted soluble factors and extracellular matrix components [49, 50]. The bone marrow is known to provide a rich stem cell niche, particularly for hematopoietic stem cells (HSCs) [49, 51] and OPN has recently been shown to be integral in supporting the HSC niche in bone by promoting the migration and lodging of HSCs within the bone endosteal region [52]. Similar to healthy stem cells within the normal bone marrow niche, stem-like cancer cells have also been shown to be influenced and maintained by components of the microenvironment in which they reside, such as stromal cells and soluble factors [53]. Recent work has implicated OPN in the maintenance of “stemness” in different types of cancers. Pietras et al (2014) showed that OPN was able to promote stemness in proneural glioblastoma [54], and Cao et al (2015) showed that OPN promotes a cancer stem cell-like phenotype in hepatocellular carcinoma cells. The ability of bone-derived OPN to maintain the stem-like behavior of breast cancer cells *in vitro* suggest that it may also support the establishment of breast cancer metastases in the bone *in vivo*.

Given our work showing that bone-derived OPN promotes the migration and stem-like behavior of breast cancer cells, we wanted to investigate which cell surface receptors bone-derived OPN uses to influence breast cancer cell properties, and we chose to evaluate the role of CD44 and RGD-dependent integrins in these functions. CD44 plays an important role in metastasis by participating in a variety of signaling networks that promote migration, invasion, growth and survival [55] and our lab has recently showed that breast cancer cells preferentially migrate toward lung-derived OPN in a CD44-dependent manner [20]. RGD-dependent cell surface integrins (α5β1, α8β1 and all αv-containing integrins) assist in adhesion and migration by recognizing ligands that contain the RGD motif (Arg-Gly-Asp), mainly found in components of the extracellular matrix such as OPN, fibronectin, and vitronectin [56].

Our results show that blocking CD44 or RGD-dependent integrins on the surface of breast cancer cells abrogates migration toward BMCM. Interestingly, we found cell line-specific migratory responses when the OPN-CD44 or the OPN-RGD interactions are blocked. MDA-MB-231 cells seem to be more dependent on CD44 versus RGD integrins for their response to bone-derived OPN; whereas SUM-159 cells depend more heavily on RGD integrins for this response. Flow cytometry analysis revealed that both cell lines express significant amounts of CD44 and similarly moderate amounts of RGD-dependent β1, αvβ3 and αvβ5 and RGD-independent α9β1, suggesting that the differences in migratory function observed could be dependent on the expression of other cell surface receptors including integrins such as α5β1 and α8β1.

Finally, we wanted to begin to explore the downstream signaling pathways that OPN activates to influence breast cancer cell properties and functions. We observed that bone-derived OPN specifically promoted the phosphorylation of WNK-1 and PRAS40, and that these pathways are important for mediating the migration of breast cancer cells in response to BMCM. WNK1 is a seronine-threonine protein kinase that is involved in the MAPK cascade and in EGF-dependent stimulation of ERK1/2 and ERK5 [57, 58]. Previous studies have shown that knockdown of WNK1 in leads to the suppression of ERK1/2 by EGF and results in reduced cell growth and migration [57]. WNK1 is also known suppress TGF-β signaling. TGF-β is integral in inducing the epithelial-to-mesenchymal transition (EMT) thus loss or inactivation of WNK1 activity could promote EMT of epithelial tumor cells [59]. WNK1 is linked to Rho GTPases, which control the dynamics of the cytoskeleton and are integral in cell migration and invasiveness [60, 61]. Our data support this role for WNK1, as knockdown of WNK1 resulted in reduced migration of breast cancer cells in response to BMCM. WNK1 is also a substrate of Akt, suggesting that it may play a role in the PI3K/Akt pathway [58]. Its role in this pathway could cause WNK1 to be indirectly phosphorylated by the interaction of bone-derived OPN with αvβ3 on breast cancer cells, and thus could contribute to breast cancer migration, invasiveness and cell growth downstream of OPN. PRAS40 is a proline-rich substrate of Akt and mTORC1 that acts at the intersection of the Akt and mammalian target of rapamycin (mTOR) signaling pathways. The expression of phospho-PRAS40 is upregulated in 40% of primary breast cancer samples and correlates with PI3K-Akt signaling and activation [33]. Based on our results, it is likely that bone-derived OPN causes phosphorylation of PRAS40 indirectly by activating the PI3K pathway since OPN signals through this pathway via αVβ3 to promote migration, invasion, cell survival and proliferation [62]. While experimental studies have demonstrated a role for the PI3K pathway in bone metastasis of breast cancer [63, 64], to the best of our knowledge neither WNK1 or PRAS40 have previously been specifically implicated in bone metastasis. Our results support the need for future studies aimed at a detailed investigation of the role of these pathways and their potential as therapeutic targets for breast cancer bone metastasis.

In conclusion, this study shows that bone-derived OPN promotes the migration of breast cancer cells and contributes to maintaining the stem-like behavior of breast cancer cells through interactions with CD44 and RGD-dependent integrins. This study also shows that bone-derived OPN activates a downstream signaling response in breast cancer cells involving the phosphorylation of WNK-1 and PRAS40, and that these pathways contribute to enhanced migration of breast cancer cells in the context of the soluble bone microenvironment. While the cancer research community has known about specific metastatic patterns and organ preferences that breast cancers exhibit for over a century, only recently have efforts focused on the role of the secondary site or “soil” in promoting and supporting metastatic growth at these sites. It is becoming increasingly clear that the bone microenvironment offers an optimal niche for the establishment and progression of metastases. Our study shows that bone-derived soluble factors–specifically OPN–contribute to this process, possibly by interacting with stem-like breast cancer cells. Future studies should concentrate on further elucidating the crosstalk between the “seed” and “soil” during metastasis as this knowledge could direct the development of novel therapeutics aimed at preventing and treating metastasis.

## Supporting information

S1 Fig Strategy for isolation of ALDH^hi^CD44^+^CD24^-^ and ALDH^lo^CD44^-^CD24^+^ breast cancer cell subpopulations. MDA-MB-231 human breast cancer cells were labeled with anti-CD44-APC, anti-CD24-PE, the ALDEFLUOR^TM^ assay kit, and 7-AAD viability dye. Cell subsets were isolated using a four-colour protocol on a FACS ARIA III. (A) Cells were first isolated based on expected light scatter and then (B) viability based on 7-AAD exclusion. (C) Cells are then analyzed for ALDH activity; the top 20% most positive for ALDH activity are deemed ALDH^hi^ and the bottom 20% for lowest ALDH activity are deemed ALDH^lo^. (D) ALDH^hi^ cells are selected for CD44^+^/CD24^-^ phenotype and (E) ALDH^lo^ cells are selected for CD44^-^/CD24^+^ phenotypes.
(TIF)

S2 Fig MDA-MB-231 human breast cancer cells express CD44 and multiple different cell surface integrins. Representative histograms are shown from flow cytometry characterization of MDA-MB-231 cells incubated with (A) PE-conjugated anti-CD44, (B) AlexaFluor-488-conjugated anti-β1 integrin, (C) FITC-conjugated anti-αvβ3 integrin, (D) AlexaFluor-488-conjugated anti-αvβ5 integrin or (E) AlexaFluor-488 conjugated anti-α9β1 integrin antibodies for 1 hour at 4°C compared to cells incubated with an isotype-matched IgG-control. Samples were run on a Beckman-Coulter EPICS XL-MCL flow cytometer (N = 3).
(TIF)

S3 Fig SUM-159 human breast cancer cells express CD44 and multiple different cell surface integrins. Representative histograms are shown from flow cytometry characterization of MDA-MB-231 cells incubated with (A) PE-conjugated anti-CD44, (B) AlexaFluor-488-conjugated anti-β1 integrin, (C) FITC-conjugated anti-αvβ3 integrin, (D) AlexaFluor-488-conjugated anti-αvβ5 integrin or (E) AlexaFluor-488 conjugated anti-α9β1 integrin antibodies for 1 hour at 4°C compared to cells incubated with an isotype-matched IgG-control. Samples were run on a Beckman-Coulter EPICS XL-MCL flow cytometer (N = 3).
(TIF)

S1 Table Metastasis-associated proteins identified in bone marrow-conditioned media with the RayBio^®^ Biotin label-based mouse antibody array. (DOCX)
