# Supplementary material for: Soluble bone-derived osteopontin promotes migration and stem-like behavior of breast cancer cells
Source: PLoS One. 2017 May 12;12(5):e0177640. doi: 10.1371/journal.pone.0177640 (PMC5428978; doi:10.1371/journal.pone.0177640)
Supplement: S1 Table — (DOCX) [file pone.0177640.s004.docx]

S1 Table: Metastasis-associated proteins identified in bone marrow-conditioned media with the RayBio^®^ Biotin label-based mouse antibody array.

| **Protein Name** | **Function/Involvement in Malignancy** | **References** |
| --- | --- | --- |
| Basic fibroblast growth factor (bFGF) | - Overexpressed in breast cancer patients - Implicated in osteoblastic bone metastases - Regulates neoplastic cell growth | [1] |
| Intracellular adhesion molecule-1 (ICAM-1) | - Elevated in metastatic breast cancer patients - Downregulation leads to a strong suppression of breast cancer cell invasion | [1] |
| Insulin-like growth factor-2 (IGF-2) | - Up-regulated in triple-negative breast cancer - Down-regulation of bone-derived IGF-2 suppresses the growth of bone metastases in prostate cancer | [3,4] |
| Matrix-metalloproteinase-14 (MMP-14) | - Modulates migratory ability of breast cancer cells - High levels of MMP-14 correlate with bone metastases | [5] |
| Osteopontin (OPN) | - Most abundant extracellular matrix protein in the bone - Associated with multiple steps of the metastatic cascade, including proliferation, migration, adhesion and invasion | [6-8] |

**S1 Table References**

1. Granato AM, Nanni O, Falcini F, Folli S, Mosconi G, De Paola F, et al. Basic fibroblast growth factor and vascular endothelial growth factor serum levels in breast cancer patients and healthy women: useful as diagnostic tools? Breast Cancer Res. 2004;6: R38-45.
2. Rosette C, Roth RB, Oeth P, Braun A, Kammerer S, Ekblom J, Denissenko MF. Role of ICAM1 in invasion of human breast cancer cells. Carcinogenesis. 2005;26: 943-950.
3. Kimura T, Kuwata T, Ashimine S, Yamazaki M, Yamauchi C, Nagai K, et al. Targeting of bone-derived insulin-like growth factor-II by a human neutralizing antibody suppresses the growth of prostate cancer cells in a human bone environment. Clin. Cancer Res. 2010;16: 121-129.
4. Hamilton N, Marquez-Garban D, Mah V, Fernando G, Elshimali Y, Garban H, et al. Biologic Roles of Estrogen Receptor- beta and Insulin-Like Growth Factor-2 in Triple-Negative Breast Cancer. Biomed. Res. Int. 2015;2015: 925703.
5. Tobar N, Avalos MC, Mendez N, Smith PC, Bernabeu C, Quintanilla M, et al. Soluble MMP-14 produced by bone marrow-derived stromal cells sheds epithelial endoglin modulating the migratory properties of human breast cancer cells. Carcinogenesis. 2014;35: 1770-1779.
6. Shevde LA, Das S, Clark DW, Samant RS. Osteopontin: an effector and an effect of tumor metastasis. Curr Mol Med. 2010;10: 71-81.
7. Tuck AB, Chambers AF. The role of osteopontin in breast cancer: clinical and experimental studies. J Mammary Gland Biol Neoplasia. 2001;6: 419-429.
8. Adwan H, Bauerle T, Najajreh Y, Elazer V, Golomb G, Berger MR. Decreased levels of osteopontin and bone sialoprotein II are correlated with reduced proliferation, colony formation, and migration of GFP-MDA-MB-231 cells. Int. J Oncol. 2004;24: 1235-1244.
